# ACGAN-Based Multi-Target Elevation Estimation with Vector Sensor Arrays in Low-SNR Environments

**DOI:** 10.3390/s25216581

**Published:** 2025-10-25

**Authors:** Biao Wang, Ning Shi, Yangyang Xie

**Affiliations:** Ocean College, Jiangsu University of Science and Technology, Zhenjiang 212100, China; wangbiao@just.edu.cn (B.W.);

**Keywords:** direction-of-arrival estimation (DOA), Auxiliary Classifier Generative Adversarial Network (ACGAN), Multi-scale Dilated Feature Aggregation (MDFA), vector hydrophone

## Abstract

To mitigate the reduced accuracy of direction-of-arrival (DOA) estimation in scenarios with low signal-to-noise ratios (SNR) and multiple interfering sources, this paper proposes an Auxiliary Classifier Generative Adversarial Network (ACGAN) architecture that integrates a Squeeze-and-Excitation (SE) attention mechanism and a Multi-scale Dilated Feature Aggregation (MDFA) module. In this neural network, a vector hydrophone array is employed as the receiving unit, capable of simultaneously sensing particle velocity signals in three directions $\left(v_{x},v_{y},v_{z}\right)$ and acoustic pressure p, thereby providing high directional sensitivity and maintaining robust classification performance under low-SNR conditions. The MDFA module extracts features from multiple receptive fields, effectively capturing cross-scale patterns and enhancing the representation of weak targets in beamforming maps. This helps mitigate estimation bias caused by mutual interference among multiple targets in low-SNR environments. Furthermore, an auxiliary classification branch is incorporated into the discriminator to jointly optimize generation and classification tasks, enabling the model to more effectively identify and separate multiple types of labeled sources. Experimental results indicate that the proposed network is effective and shows improved performance across diverse scenarios.

## 1. Introduction

Direction-of-arrival (DOA) estimation [1] is widely applied in numerous research areas such as underwater target detection, array signal processing, sound source localization, radar systems, and intelligent mobile positioning. Traditional DOA algorithms, such as Multiple Signal Classification (MUSIC) [2] and Estimation of Signal Parameters via Rotational Invariance Techniques (ESPRIT) [3], achieve high-precision direction estimation by constructing covariance matrices and performing eigen-decomposition. Reduced-rank Covariance Matrix (RCM) methods [4,5] improve the stability of the covariance matrix under limited snapshot conditions by constructing optimal low-rank subspaces, outperforming conventional methods like MUSIC in signal separation and interference suppression. An approach integrating coprime array interpolation with MUSIC was proposed in [6] to mitigate false source interference during rank reduction. In [7], a virtual signal array was constructed to exploit spatial information from sparse arrays, thereby enhancing estimation accuracy and resolution. A high-dimensional DOA algorithm based on coarray tensor modeling was introduced in [8], which improves source identification through tensor decomposition. However, these methods may suffer from degraded stability under extreme noise, structural uncertainty, or non-ideal source models. Recent research shows that deep learning has become a powerful tool in DOA estimation. In [9], a deep learning-based co-prime array interpolation method was proposed to fill virtual array a hole and enhance DOA estimation accuracy. In [10], a multi-task autoencoder and classification strategy were used to effectively extract directional features, though the model lacked generative capability and attention mechanisms. In [11], deep neural networks were trained directly on covariance matrices, comparing multiple architectures with different input forms, and showing that convolutional neural networks (CNNs) outperform other methods under low signal-to-noise ratios (SNR) conditions. A CNN-based model was proposed in [12], which learns DOA information directly from spatiotemporal signals and reformulates the DOA task as an image classification problem, avoiding reliance on covariance matrices and subspace methods. DeepMUSIC [13] combined the classical MUSIC algorithm with a deep CNN architecture. In [14], a CNN-based method incorporated a sparse prior during training to enhance the sparsity of the angular distribution. A multi-label classification model using logarithmic eigenvalue features was proposed in [15]. An end-to-end 2D DOA estimation framework combining dual 1D CNNs with deep learning was developed in [16]. In [17], optimized short-time Fourier transform parameters combined with a temporal convolutional network significantly improved precision and F1-score in moving sound source localization. Building on GAN architectures, a DCGAN-based method was developed in [18] to address gridless and underdetermined DOA estimation scenarios. In [19], a super-resolution DOA network (SDOA-Net) utilizing raw received signals instead of covariance matrices was developed to improve estimation robustness for imperfect arrays. Despite notable advancements in the field of DOA estimation, most studies have focused on azimuth estimation in 2D planes. In fact, in practical applications underwater source localization, radar monitoring, and 3D imaging, elevation angle is equally critical for spatial positioning. However, elevation modeling remains underexplored, and dedicated neural architectures for elevation estimation are still lacking.

To address the challenges of false source interference and poor target discrimination in multi-target elevation estimation under low-SNR conditions, we introduce a deep learning model derived from the Auxiliary Classifier Generative Adversarial Network (ACGAN) architecture to tackle this challenge. By leveraging adversarial interactions between the generator and discriminator, the model achieves greater robustness in handling small datasets and irregular noise environments. Furthermore, a novel Multi-scale Dilated Feature Aggregation (MDFA) module is integrated into the network architecture to improve multi-scale spatial modeling of elevation features. This module significantly suppresses background interference and highlights directionally discriminative features. The proposed model offers three key advantages: (1) accurate modeling of multi-target elevation structures; (2) strong robustness against non-uniform noise; and (3) high discrimination accuracy under low-SNR conditions.

The remainder of this paper is organized as follows: Section 2 presents the signal model for elevation estimation; Section 3 details the network architecture, including the generator, discriminator, and loss function design; Section 4 provides experimental results and performance analysis; Section 5 discusses the findings; and Section 6 concludes the paper.

## 2. Signal Model

**Figure 1:**
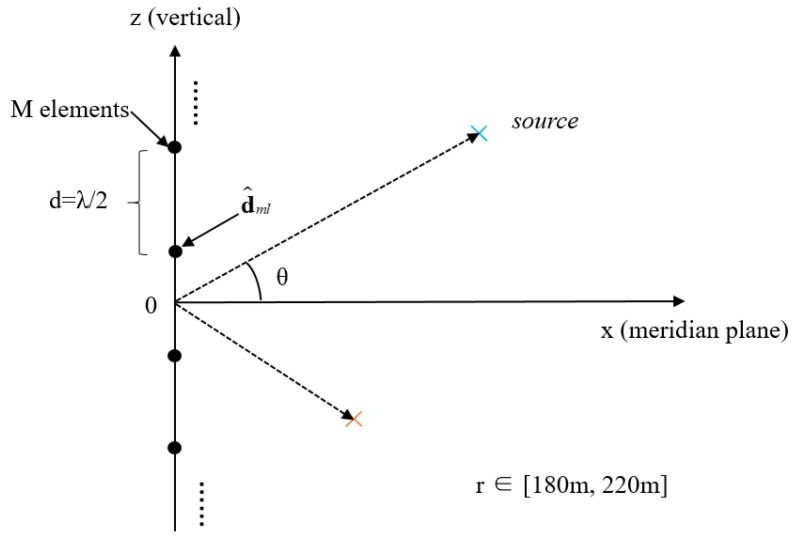
Schematic illustration of the array and sound sources. The black dots represent the hydrophone elements of the vertical array, while the blue and orange crosses denote the sound sources located at different elevation angles.

In this study, it is assumed that the sources and the array lie in the same two-dimensional plane (the x–z plane, i.e., y = 0). A vertical linear array composed of M vector hydrophones is constructed to receive signals from L far-field narrowband sources, assuming L<M. The received signal at time t is given by

(1) $$x(t)=\sum_{l=1}^{L}a(\theta_{l})s_{l}(t-\tau_{l})+n(t)=A(\theta )\cdot s(t)+n(t)$$

where $s(t)={\left[s_{1}(t),\ldots ,s_{L}(t)\right]}^{T}$ denotes the signal vector from L; $a(\theta_{l})$ is the array manifold vector corresponding to the l-th signal incident at angle $\theta_{l}$; $A=[a(\theta_{1}),\ldots ,a(\theta_{L})]\in {\mathbb{C}}^{M\times L}$; n(t) represents zero-mean complex Gaussian noise; $\tau_{l}\;$ is the propagation delay from the l-th source to the receivers. Each source is located in space by its elevation angle θ∈[−90°,90°] and distance r∈[180 m,220 m]. To better illustrate the spatial geometry, a schematic diagram of the array and sources is provided (see Figure 1, which clearly shows the vertical array structure, hydrophone spacing, source positions, elevation angle θ, and propagation direction vector ${\hat{d}}_{ml}$. The propagation delay from the source to the m-th sensor is calculated based on 3D geometry as

(2) $$\tau_{m}^{\left(l\right)}=\frac{\left\|r_{m}-r_{l}\right\|}{c}$$

where $r_{l}$ is the coordinate of the lll-th source in the two-dimensional plane (x–z plane, y = 0), and $r_{m}$ is the position of the m-th sensor. In this study, a two-dimensional approximation (x–z plane, y = 0) is adopted, so that both $r_{l}$ and $r_{m}$ represent coordinates within this plane. Although the formulas are written in a 3D form, they are actually constrained to the 2D plane.

Signal attenuation during propagation is modeled using an exponential decay:

(3) $$\alpha_{m}^{\left(l\right)}=0.5+0.5\cdot e^{-d_{ml}/200}$$

where $d_{ml}$ is the distance between the l-th source and the m-th receiver.

In addition to traditional acoustic pressure signals $p_{l}(t)$, the model also considers the three-dimensional particle velocity components $\left(v_{x}^{\left(l\right)},v_{y}^{\left(l\right)},v_{z}^{\left(l\right)}\right)$ from each source. The total received signal consists of the superposition of multiple sources and noise. For the l-th source, the particle velocity can be approximated as the product of the time derivative of the pressure and the propagation direction:

(4) $$v_{m}^{\left(l\right)}(t)=-\frac{1}{\rho c}\frac{\partial p_{l}(t-\tau_{m}^{\left(l\right)})}{\partial t}{\hat{d}}_{ml}$$

For all sources, the total particle velocity at the m-th sensor is:

(5) $$v_{m}(t)=\sum_{l=1}^{L}v_{m}^{\left(l\right)}(t)+n_{v}(t)$$

where ${\hat{d}}_{ml}$ denotes the unit direction vector from the l-th source to the m-th sensor, and $\rho =1000\;\mathrm{kg}/m^{3}$ is the density of water.

In this study, we assume the vector hydrophones to be ideal omnidirectional sensors: the pressure channel is omnidirectional, while the three orthogonal particle velocity channels also have ideal directional responses. Four vector hydrophone channel combinations are used in the experiments: Mode 1: pressure only p; Mode 2: $p+v_{z}$; Mode 3: $v_{x}+v_{y}+v_{z}$; Mode 4: $p+v_{x}+v_{y}+v_{z}$. All temporal signals received at each sensor are transformed using a 128-point Fast Fourier Transform (FFT), and only the first half of the spectrum is retained. The real and imaginary parts of the spectrum are used as features.

To simulate non-ideal conditions, additive Gaussian noise with varying SNRs is applied independently to each channel and sensor. The SNR values are randomly selected from the range [−10 dB,+10 dB], and the corresponding noise power is computed as

(6) $$\sigma^{2}=\frac{P_{signal}}{10^{SNR/10}}$$

This noise modeling approach better reflects real-world conditions, especially for elevation angle estimation in non-uniform and complex acoustic environments.

## 3. Network Model

### 3.1. Overall Network Architecture Design

**Figure 2:**
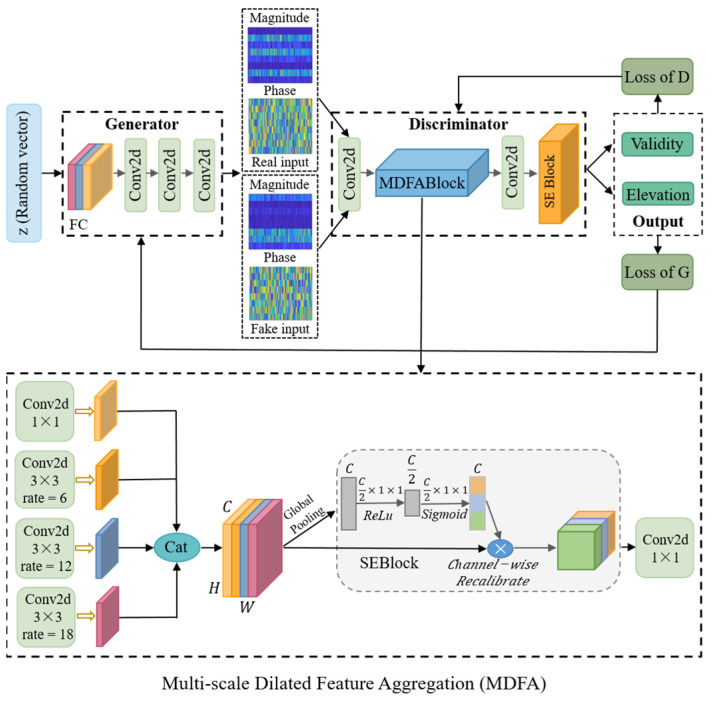
Overall Network Architecture Design. The colored blocks represent different functional components of the model: blue blocks indicate the MDFA module, and orange blocks correspond to the SE attention block. The magnitude and phase inputs are shown as color-mapped feature matrices.

As depicted in Figure 2, the proposed model for DOA estimation is designed based on an ACGAN structure. The proposed framework integrates two primary components, namely the Generator and the Discriminator, which are collaboratively optimized through an end-to-end training process guided by a composite loss. The Generator receives a random noise vector and transforms it into synthetic features that mimic the frequency characteristics of real-world signals. The Discriminator, upon receiving these frequency-domain features, performs two tasks simultaneously: (1) determining the authenticity of the input samples (validity), and (2) predicting the corresponding elevation angle class labels. By jointly optimizing both adversarial and classification objectives, the model progressively improves its robustness and representation capability, particularly in low-SNR and non-uniform noise environments.

To enhance the model’s perception of complex signal patterns, this study introduces a novel MDFA module into the discriminator, which is jointly modeled with a squeeze-and-excitation (SE) attention mechanism. This combination improves the model’s discrimination capability under low-SNR and multi-target conditions. The generator consists of fully connected layers followed by multiple convolutional modules and is designed to produce multi-channel frequency-domain feature maps with both magnitude and phase components, aiming to deceive the discriminator. The discriminator is composed of an initial convolutional block, the MDFA module, and the SE attention block, and performs two tasks through dual output branches: real/fake discrimination and elevation angle classification. During training, adversarial learning is carried out through the competition between the generator and the discriminator. The discriminator is optimized using a combination of adversarial loss and classification loss, thereby improving its ability to distinguish between real and fake signals as well as to classify elevation angles. The generator, in turn, is trained to generate high-quality feature maps that are misclassified as real and assigned the correct angle labels by the discriminator. Details of the composite loss function are provided in Section 3.4.

In summary, the proposed model builds on the ACGAN framework by integrating multi-scale feature extraction and attention mechanisms, significantly enhancing its elevation estimation performance under adverse conditions such as low-SNR, multi-target scenarios, and non-uniform noise.

### 3.2. The Architecture of the Generator

In the ACGAN model, the generator is designed to transform random noise vectors into frequency-domain feature maps that mimic real signals. This process provides the discriminator with a large number of diverse training samples, thereby enhancing sample diversity and enabling the discriminator to perform more stable and accurate signal identification and elevation angle estimation.

The input to the generator is a random vector $z\in R^{100}$, sampled from a standard normal distribution. This vector is first projected through a fully connected (Dense) layer into a high-dimensional tensor that matches the target shape. The output tensor has the same dimensionality as the input to the discriminator, i.e., M×T×C, where M represents the number of sensors, T indicates the temporal resolution, and C accounts for both real and imaginary parts. The high-dimensional output is then reformatted into a three-dimensional tensor of shape (M,T,C). After stacking B such samples, the final output tensor becomes four-dimensional with shape (B,M,T,C), where B is the batch size. This output tensor represents the generated complex-valued frequency-domain feature maps, which are designed to simulate the frequency characteristics of real signals.

The Generator does not include any upsampling or convolutional stacking modules, thereby maintaining a lightweight architecture that accelerates model training. During training, the Generator gradually learns the distribution of real data through the joint optimization of adversarial and classification losses. Its objective is to maximize the discriminator’s error probability when distinguishing fake samples, ensuring that the generated outputs not only successfully fool the discriminator but also carry explicit elevation angle information. It enhances the discriminator’s ability to identify authentic versus synthetic signals and supports the learning of informative features. This mechanism significantly improves the network’s overall robustness and generalization ability for elevation angle estimation, particularly under conditions of sample imbalance or low-SNR.

### 3.3. The Architecture of the Discriminator

The discriminator is a critical component of the ACGAN framework, responsible for two primary tasks: (1) determining whether the input feature map is real or fake, and (2) classifying the elevation angle of the input sample. This module is required to perform robustly during training, as it must not only possess strong feature extraction capabilities to capture meaningful patterns from frequency-domain structures but also maintain high robustness under challenging conditions such as low-SNR, multiple targets, and non-uniform noise. The Discriminator takes as input either real signals or “fake” samples generated by the generator. These inputs are complex-valued frequency-domain feature maps comprising array spatial dimensions, temporal dimensions, and real–imaginary channels. First, the input feature map is processed by two convolutional blocks, each consisting of a 2D convolutional layer, a Leaky ReLU activation, and batch normalization, to extract low-level local features. The resulting feature maps are then passed through an MDFA module. This module employs parallel convolutional paths with different dilation rates to extract multi-scale features, which are then concatenated and fused to enhance the model’s perception of spatial structures at various scales. Next, a convolutional layer is applied to compress and integrate the spatial features, followed by a channel attention mechanism implemented using an SE block. The SE module captures global contextual information and guides the discriminator to focus on feature channels that are highly relevant to elevation angle estimation, while suppressing redundant interference and non-uniform noise. Finally, the refined feature map undergoes global average pooling to produce a one-dimensional vector, which is fed into two output branches. One branch uses a sigmoid activation function to output the probability that the sample is real or fake, while the other branch employs a softmax function to produce the probability distribution over elevation angle classes. Through the coordinated integration of convolutional feature extraction, multi-scale dilated perception, and attention-based channel refinement, the discriminator achieves enhanced discrimination and classification performance under complex acoustic conditions. Moreover, it provides more informative feedback to the Generator during adversarial training.

#### 3.3.1. Summary of the Channel Attention Mechanism

The fundamental purpose of attention mechanisms [20] is to enhance salient features while suppressing irrelevant or interfering signals. In the field of computer vision, attention-based methods have shown great success across tasks such as classification, detection, and segmentation by significantly improving performance. Among them, channel attention mechanisms play a particularly important role. They aim to extract global context by compressing feature maps along the channel dimension and modeling inter-channel dependencies, thereby highlighting informative channels and suppressing redundant information. The SE module proposed by Hu et al. [21] is one of the most representative implementations and is now commonly used as a standard form of channel attention. SE modules and their variants have been extensively applied across a range of tasks. For instance, in [22], SE attention was adapted for array signal modeling, where Liu et al. proposed the Array Covariance Attention (ACA) mechanism tailored to enhance DOA estimation under nonuniform noise conditions. In [23], cross-attention was combined with graph convolution networks. In [24,25,26], SE modules were integrated with the YOLO framework to enhance feature fusion, detection accuracy, and robustness; In [27,28], SE attention was embedded into domain-specific signal modeling modules. However, most existing SE-based mechanisms focus primarily on channel-wise weighting and may struggle to capture complex interactions among multi-scale and multi-target features, especially in low-SNR scenarios. To address this limitation, we propose an MDFA module that integrates SE attention with a multi-scale dilated convolutional structure, enabling more effective feature aggregation and discrimination under challenging conditions.

#### 3.3.2. Structure and Principle of Multi-Scale Dilated Feature Aggregation

**Figure 3:**
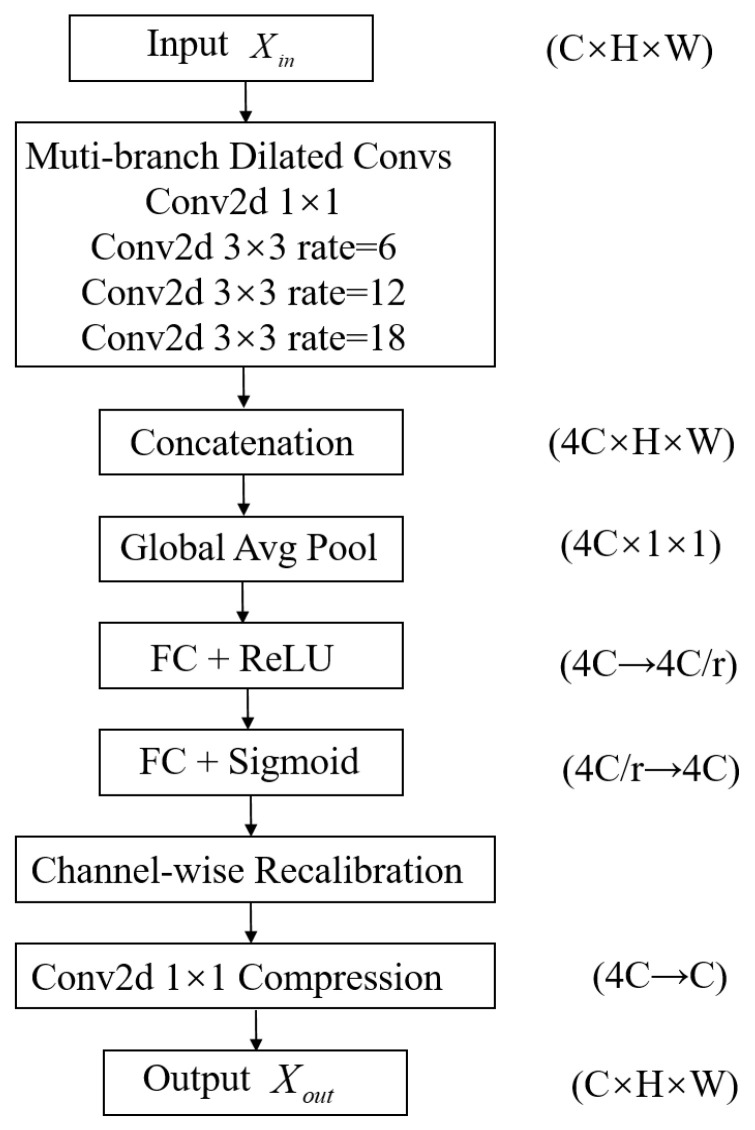
Flowchart of the MDFA Module.

The MDFA module consists of four main steps: multi-scale dilated feature extraction, feature concatenation and fusion, channel attention weighting, and convolutional integration for final output. The complete computational flow is illustrated in Figure 3.

Step 1: Multi-Scale Dilated Feature Extraction

The MDFA module employs four parallel convolutional paths to extract spatial contextual information from the input feature map. These branches include one standard convolution with consistent channel dimensions to preserve local fine-grained information, and three dilated convolutions with dilation rates of 6, 12, and 18 to extract long-range dependencies. Let the output feature map from the i-th convolutional branch be denoted as $X_{i}$. The multi-branch extraction results can thus be represented as

(7) $$\left\{X_{1},X_{2},X_{3},X_{4}\}={\mathrm{Conv}}_{\mathrm{dilated}}^{\left(i\right)}(X\right)$$

Step 2: Feature Concatenation and Fusion

To generate a fused representation, the four outputs from different receptive fields are combined through channel-wise concatenation:

(8) $$X_{\mathrm{concat}}=\mathrm{Concat}(X_{1},X_{2},X_{3},X_{4})\in R^{C^{\prime }\times H\times W}$$

where $C^{\prime }=4C$. The fusion operation retains both local and global structural information, providing more comprehensive input for subsequent attention mechanisms and enhancing the model’s ability to allocate attention weights for feature discrimination.

Step 3: Channel Attention Weighting

To enhance feature discriminability, the fused feature map is passed through a SE module, which performs three steps—squeeze, excitation, and reweighting—to strengthen important features and suppress irrelevant ones.

Squeeze: To generate a compressed representation, global average pooling is performed on each channel:

(9) $$Z_{c}=\frac{1}{H\times W}\sum_{i=1}^{H}\sum_{j=1}^{W}x_{\mathrm{concat},c}(i,j)$$

The outputs across all channels are assembled into a vector $Z\in R^{C^{\prime }\times 1\times 1}$.

Excitation: To encode channel-wise interactions, Z is transformed through two fully connected layers activated by ReLU and sigmoid functions:

(10) $$\hat{Z}=\sigma (W_{2}\cdot \mathrm{ReLU}(W_{1}\cdot Z))$$

where $W_{1}\in R^{\frac{C^{\prime }}{r}\times C^{\prime }}$ and $W_{2}\in R^{C^{\prime }\times \frac{C^{\prime }}{r}}$, with r denoting the reduction ratio (default value: 16).

Reweighting: The attention weights $Z\in R^{C^{\prime }\times 1\times 1}$ are applied to the fused feature map to obtain the weighted features:

(11) $$\hat{X}=X_{\mathrm{concat}}\cdot \hat{Z}$$

This attention mechanism helps suppress redundant or noisy channels and emphasizes critical directional information.

Step 4: Convolutional Integration and Output

To obtain the final feature output, a 1×1 convolution is applied to the reweighted feature map $\hat{X}$, adjusting the channel dimensions accordingly:

(12) $$X_{\mathrm{out}}={\mathrm{Conv}}_{1\times 1}(\hat{X})$$

Module Advantage: Compared to traditional SE-based attention mechanisms, the proposed MDFA module incorporates multi-scale dilated convolutions, which significantly expand the effective receptive field without increasing parameter overhead. By capturing spatial features from local to global scales, the model also learns to represent the intricate positional dependencies across multiple targets. In scenarios with low-SNR or high target overlap, the MDFA module significantly improves the recognition and robustness of directional features.

### 3.4. Loss Function Design

To achieve joint optimization of adversarial learning and multi-target elevation estimation, this study constructs a composite loss function framework based on the ACGAN architecture. The proposed framework takes into account factors such as label distribution, class imbalance, confidence regularization, and generator-discriminator stability. The overall design is as follows:

(1) Kullback–Leibler (KL) Divergence Loss

The model adopts KL divergence as the primary loss function to compare predicted and target distributions, as the elevation labels are provided in the form of Gaussian-smoothed soft annotations:

(13) $$L_{\mathrm{KL}}=\sum_{i}y_{i}\cdot \log(\frac{y_{i}}{{\hat{y}}_{i}+\epsilon })$$

where $y_{i}$ and ${\hat{y}}_{i}$ denote the target and predicted distributions, respectively, and ϵ is a small constant added for numerical stability.

(2) Focal Loss

Focal loss is incorporated to help the model focus on low-confidence and hard-to-classify samples:

(14) $$L_{\mathrm{focal}}=-{\left(1-m\right)}^{\gamma }\cdot \log(m+\epsilon )$$

where $m=\sum_{i}y_{i}{\hat{y}}_{i}$ represents the similarity between predicted and target distributions, and γ=2.0 controls the attention to hard samples.

(3) L1 Distribution Loss

To complement the KL loss and improve precision in multi-target output distribution, the L1 loss is introduced as

(15) $$L_{L1}=\sum_{i}\left|y_{i}-{\hat{y}}_{i}\right|$$

(4) Confidence Penalty

To suppress overconfident predictions in non-target regions and improve uncertainty modeling, a confidence penalty term based on information entropy is defined as

(16) $$L_{\mathrm{conf}}=\sum_{i}{\hat{y}}_{i}\cdot \log({\hat{y}}_{i}+\epsilon )$$

This penalizes only those outputs with high confidence in irrelevant regions, allowing the model to retain appropriate uncertainty elsewhere, thereby enhancing both prediction precision and generalization.

(5) Permutation Invariant Binary Cross-Entropy (PIT-BCE)

To address label permutation ambiguity in multi-target prediction, we employ the Permutation-Invariant Training (PIT) strategy, which explores all possible label permutations and selects the one that minimizes the binary cross-entropy loss.

(17) $$L_{\mathrm{cls}}^{\mathrm{PIT}}=\min_{\pi \in P}\sum_{i=1}^{K}w_{y_{\pi (i)}}\cdot (y_{\pi (i)}\log{\hat{y}}_{i}+(1-y_{\pi (i)})\log(1-{\hat{y}}_{i}))$$

(6) Feature Consistency Loss

A feature consistency loss is incorporated to promote structural realism in generated elevation features by enforcing similarity with real features within the classification pathways:

(18) $$L_{\mathrm{feat}}=\mathrm{MSE}(\mathrm{Softmax}(f_{\mathrm{real}}),\mathrm{Softmax}(f_{\mathrm{fake}}))$$

This loss reduces distribution distance between soft classification outputs, guiding the generator to produce higher-quality, structure-aware samples, thereby improving discriminability and training stability.

Total Loss

Finally, the overall classification loss function is formulated as

(19) $$L_{\mathrm{total}}=L_{\mathrm{KL}}+\alpha \cdot L_{\mathrm{focal}}+\beta \cdot L_{L1}+\lambda_{\mathrm{cp}}\cdot L_{\mathrm{conf}}+L_{\mathrm{cls}}^{\mathrm{PIT}}+\lambda_{\mathrm{feat}}\cdot L_{\mathrm{feat}}$$

The weights of each loss component are empirically tuned and set as follows: α=0.7, β=0.3, $\lambda_{cp}=0.01$, $\lambda_{feat}=0.2$. This composite loss integrates multiple objectives from both the generator and discriminator, significantly improving the model’s stability and classification performance under low-SNR and multi-target scenarios.

**Figure 4:**
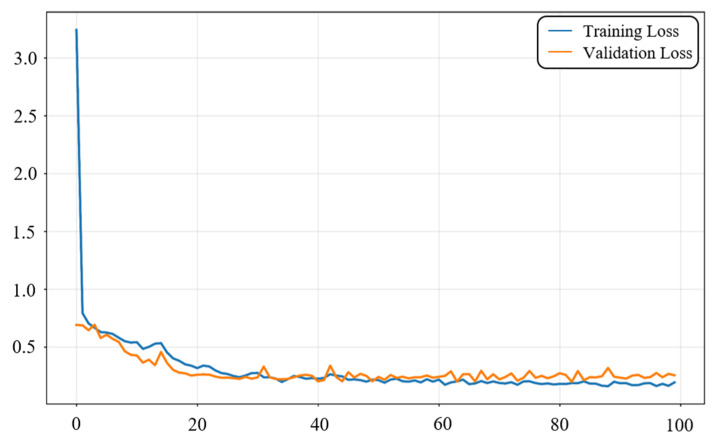
Training and validation loss during model training.

Figure 4 illustrates the variation in the composite loss function during model training. In the early stage, the training loss decreases rapidly, indicating that the model quickly captures the main features and achieves initial convergence. After 20 epochs, both training and validation losses gradually enter a stable region. Between 30 and 100 epochs, the losses remain at a low level, showing that the model achieves stable convergence after multiple iterations. During convergence, the training and validation loss curves occasionally cross, which is mainly caused by fluctuations in different mini-batches, but their overall trends remain consistent. In the later stage, the validation loss is generally higher than the training loss, reflecting that the model fits the training set more thoroughly while still maintaining good generalization ability on unseen validation data. These loss curves confirm that the proposed composite loss function can achieve stable and effective optimization under low-SNR and multi-target conditions, providing reliable support for the subsequent experiments.

### 3.5. ACGAN Training

To train and validate the proposed model, we constructed a synthetic dataset using a vertical linear array with 8 vector hydrophones (M=8). This subsection describes the dataset generation and training configurations used in the experiments. Each hydrophone recorded both acoustic pressure p and 3D particle velocity components $v_{x},v_{y},v_{z}$, forming a complex-valued multi-channel input. Three target elevation angles were configured, with simulated arrivals spanning the range [−90°,90°]. The elevation angle labels are implemented using binned classification, where the range [−90°,90°] is uniformly divided into 181 bins, each with a resolution of 1°. Although the network is trained as a classification task, the final elevation estimates are continuous, recovered from all bin centers through probability-weighted averaging (soft-argmax). This ensures higher precision than relying solely on the bin resolution. To evaluate robustness and generalization under varying SNRs and snapshot numbers, we designed a dataset covering a wide range of conditions. SNRs were set from −10 dB to 10 dB in 2.5 dB steps (9 levels in total), and snapshot numbers were set to 50, 100, 150, 250, and 500. The final dataset contained 27,000 samples, including frequency-domain features, covariance matrices, and corresponding multi-label elevation annotations. The dataset was split into 80% for training and 20% for validation. All experiments were conducted on a remote server equipped with an NVIDIA RTX 3090 Ti GPU (24 GB, NVIDIA Corporation, Santa Clara, CA, USA), an 18-core Intel^®^ Xeon^®^ E5-2696 v3 CPU (Intel Corporation, Santa Clara, CA, USA), and 64 GB of RAM. The core software stack consisted of Python 3.8, PyTorch version 2.0.0, and CUDA 11.8.0. The system was configured using an official container image provided by the platform. All deep learning models were implemented and trained using the PyTorch framework. These configurations ensure consistency across experiments and provide the foundation for the subsequent performance evaluation presented in Section 4.

## 4. Experimental Results

### 4.1. Verification of the Advantage of Vector Arrays Under Vertical Incidence

To verify the superiority of vector hydrophone arrays over traditional scalar pressure arrays in estimating elevation angles under vertical incidence conditions, we designed a dedicated comparative experiment based on our dataset. In this experiment, three target elevation angles were set, with one fixed at 90°, and the other two randomly generated within the range of [−90°,90°] but excluding 90°. The SNR was set to 0 dB, and the number of snapshots was fixed at 500. All other settings were kept consistent with the main experiment. Two input configurations were selected for comparison: one using only scalar pressure input (p); the other using full vector input $\left(p+v_{x}+v_{y}+v_{z}\right)$. To ensure statistical validity, the same model architecture and training parameters were used for both configurations. Each setup was trained and evaluated independently 100 times. The average accuracy and standard deviation were computed to evaluate performance.

Experiment 1: Performance Comparison between Vector and Scalar Arrays Under Vertical Incidence.

**Figure 5:**
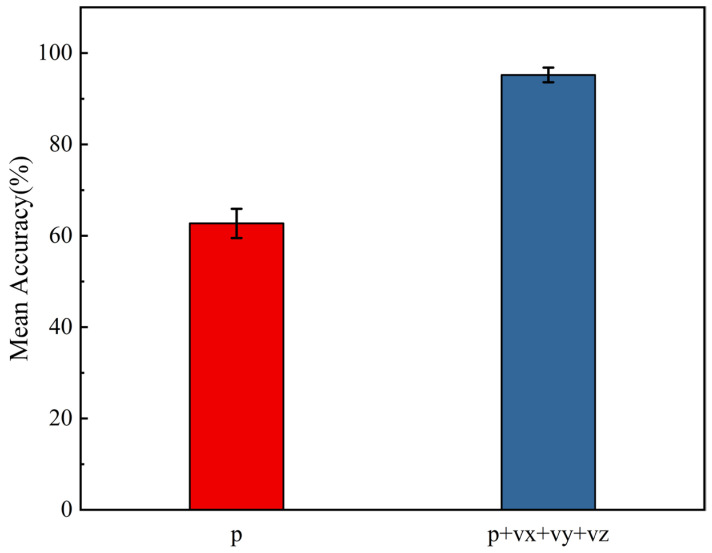
Performance comparison of different array types under vertical signal incidence.

As shown in Figure 5, the scalar hydrophone array achieved an average accuracy of only 62.7% with a standard deviation of 3.2%. This performance gap can be attributed to the fact that under vertical signal incidence, scalar hydrophones receive nearly identical acoustic pressure across elements, resulting in negligible phase differences. Consequently, elevation estimation methods based solely on pressure p become almost ineffective. In contrast, the full vector hydrophone array, which integrates multi-channel inputs $p+v_{x}+v_{y}+v_{z}$, achieved a significantly higher average accuracy of 95.2% with a standard deviation of just 1.6%, indicating superior precision and stability. This improvement benefits from the vector hydrophones’ ability to capture not only acoustic pressure but also the particle velocity induced by sound waves—particularly the z-axis component that is highly responsive to vertically incident signals. This enhancement further validates the robustness of the vector array in elevation angle estimation. The results confirm that vector hydrophones exhibit significant advantages in resolving elevation under complex incidence conditions, particularly when scalar arrays suffer from directional ambiguity. Thus, the experiment reinforces the effectiveness and adaptability of vector arrays in multi-target elevation estimation scenarios.

### 4.2. Impact of Input Channels on Elevation Angle Estimation Performance

To investigate the performance variation in elevation angle estimation under different acoustic input channel settings, this study four input channel configurations were analyzed: (1) pressure-only channel p, (2) pressure and vertical particle velocity $p+v_{z}$, (3) particle velocity components $v_{x}+v_{y}+v_{z}$, and (4) full input channels $p+v_{x}+v_{y}+v_{z}$. The pressure and particle velocity features were concatenated along the channel dimension and then fed into the model. Each input—pressure p and velocity components $v_{x},v_{y},v_{z}$—was represented by its complex-valued signal, decomposed into real and imaginary parts and concatenated by channel. For example, with the full combination $p+v_{x}+v_{y}+v_{z}$, the model receives complex features from four physical channels (each split into real and imaginary parts), resulting in eight total input channels. This approach preserves the physical distinctiveness of each input while enabling multi-source cooperative feature learning within the network. All experiments were conducted under a snapshot number T=500. The SNR varied from −10 dB to 10 dB in 2.5 dB steps, resulting in nine discrete levels. To evaluate performance across diverse noise conditions, Root Mean Square Error (RMSE) and F1-score were adopted as the primary metrics.

Experiment 2: Performance Comparison of Different Input Channels.

**Figure 6:**
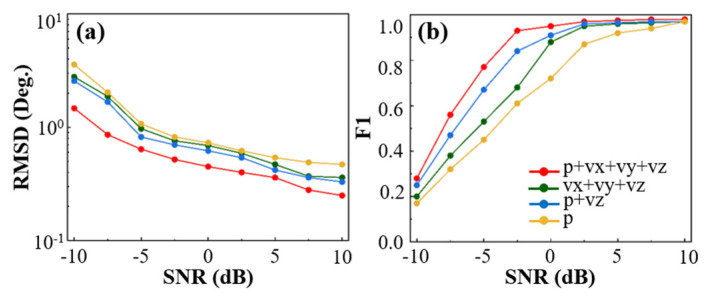
Performance comparison of different input channel configurations. (**a**) RMSE of each input configuration under varying SNR levels. (**b**) F1-score of each input configuration under varying SNR levels.

As shown in Figure 6, under the overall trend, the RMSE of all four input channel configurations gradually decreases with increasing SNR, while the F1-score steadily increases. This indicates that the model can more accurately estimate elevation angles and identify multiple targets under high-SNR conditions. However, under low-SNR conditions, the performance differences between configurations become more pronounced. When using only the pressure input p, the RMSE remains relatively high, and the corresponding F1-score at −10 dB is only 0.16, suggesting that it is difficult to accurately estimate elevation and reliably identify targets in strong noise backgrounds using only pressure information. In contrast, introducing vertical particle velocity $v_{z}$ alongside pressure $p+v_{z}$ educes RMSE and improves F1-score, demonstrating that adding velocity components enhances the model’s ability to extract spatial features. When the input is $v_{x}+v_{y}+v_{z}$, despite lacking pressure information, the model still performs better than using *p* alone in terms of both RMSE and F1-score. This suggests that multidimensional velocity inputs improve robustness to noise. Nevertheless, due to redundancy among the velocity components in a vertical array, the performance of the $v_{x}+v_{y}+v_{z}$ configuration is slightly inferior to the $p+v_{z}$ configuration. When all channels are included $p+v_{x}+v_{y}+v_{z}$, the model achieves the lowest RMSE and highest F1-scores across all SNR levels. Notably, at −10 dB, compared to using only *p*, the RMSE is reduced by more than 2.1°, and the F1-score improves by approximately 64%. These results highlight the advantage of multi-channel fusion in capturing spatial features and detecting multiple targets, even in noisy environments. The $p+v_{x}+v_{y}+v_{z}$ configuration not only provides complete spatial directional information but also offers stronger noise resistance, effectively addressing elevation estimation and detection issues in low-SNR scenarios. In summary, incorporating particle velocity information significantly enhances elevation estimation accuracy and target identification, especially when using the full-channel input configuration. The combined RMSE and F1-score improvements confirm the advantage of vector hydrophones in complex underwater acoustic environments, revealing their application potential in multi-target elevation estimation under low-SNR conditions.

### 4.3. Performance Comparison of Different Algorithms for Elevation Angle Estimation

Before comparing the performance of different algorithms for elevation angle estimation, we first clarify the experimental setup to ensure consistency and comparability of the subsequent analyses and results. The input features used in this section are based on full vector acoustic information. The input configuration combines all channels (i.e., $p+v_{x}+v_{y}+v_{z}$), which has been shown in the previous experiment to provide superior robustness and estimation accuracy, particularly under low-SNR conditions. Therefore, this configuration is adopted as the unified input format for all deep learning models in the comparative analysis. For traditional DOA estimation methods, such as MUSIC and RCM, covariance matrices are directly precomputed from the dataset. This avoids instability due to the limited number of snapshots or low-SNRs, and ensures a fair comparison in terms of input dimensionality and feature sources. For brevity in subsequent figures, the proposed multi-scale dilated feature aggregation module is abbreviated as MDFA. To reduce the influence of snapshot count on performance, the number of snapshots is fixed at T=500. In experiments with varying SNRs, the SNR is set to 0 dB to isolate its effect on estimation performance from that of snapshot number. In terms of performance metrics, three evaluation indicators are used: RMSE, F1-score, and Accuracy. Predicted angles are first matched to the ground truth using the Hungarian algorithm. RMSE is computed as the root mean square error of all matched pairs. Precision and Recall are computed under a tolerance Δθ (set to $\pm 3^{\circ }$), with the F1-score calculated as their harmonic mean. The $\pm 3^{\circ }$ tolerance threshold was determined based on the 3 dB beamwidth of the simulated array, which defines the minimum resolvable angular separation under the given configuration. A prediction is considered correct if its deviation from the ground truth is within the tolerance Δθ, and Accuracy is defined as the ratio of correct predictions. This ensures all three metrics remain consistent and comparable in multi-target scenarios.

Experiment 3: Performance Comparison of Algorithms under Different SNRs.

**Figure 7:**
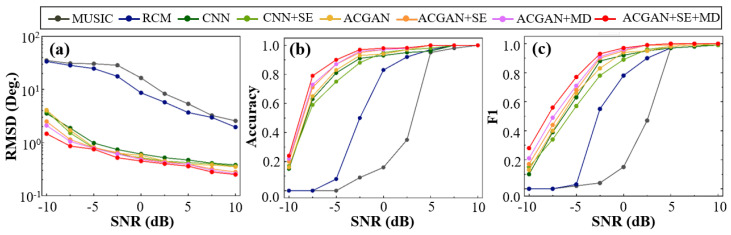
Evaluation of multiple algorithms under different SNR conditions. Subplots show (**a**) RMSE, (**b**) accuracy, and (**c**) F1-score across various noise levels.

Figure 7 illustrates the performance trends of various algorithms across different SNR levels in terms of RMSE, Accuracy, and F1-score. The detailed RMSE values of all algorithms under different SNR levels are summarized in Table 1 for quantitative comparison. Overall, with increasing SNR, all algorithms exhibit notable performance improvements, indicating their enhanced ability to detect and estimate elevation angles of multiple targets under higher SNR conditions. However, the performance gap between algorithms becomes more pronounced at lower SNRs, reflecting differences in robustness and sensitivity. As shown in Figure 7a, traditional methods such as MUSIC and RCM suffer severe performance degradation under low-SNR conditions due to their heavy reliance on the structural characteristics of covariance matrices. In particular, MUSIC fails to compute effectively in noisy scenarios. This is because its performance is highly sensitive to noise corruption in the covariance matrix, which significantly reduces angular resolution and estimation accuracy. In contrast, deep learning-based approaches demonstrate stronger adaptability, especially the proposed ACGAN + SE + MD model, which consistently outperforms others across all SNR levels, particularly under low-SNR conditions. Low-SNR scenarios often involve significant spectral overlap among multiple targets, which hampers the ability of conventional convolutional structures to distinguish responses from different directions. In this context, the MDFA module within ACGAN enables extraction of elevation-relevant features at multiple spatial scales, enhancing the model’s capacity to capture subtle but crucial distinguishing cues. This suppresses feature blurring and thereby improves estimation accuracy. The superior performance is not merely attributed to the deep learning framework itself, but rather to the structural design of ACGAN + SE + MD, which exhibits strong adaptability to multi-target elevation estimation tasks. As shown in Figure 7b, when SNR < 0 dB, traditional methods yield poor classification accuracy. In contrast, deep learning models can autonomously learn complex pattern recognition rules through end-to-end training, achieving an average accuracy above 0.9. As the SNR increases beyond 0 dB, the accuracy of all models gradually approaches 1. Notably, the ACGAN + SE + MD model, by leveraging multi-scale channels and classifier fusion, consistently achieves the highest accuracy. Figure 7c further supports this finding, highlighting the superior F1-score of ACGAN + SE + MD in multi-target classification tasks. Under −10 dB conditions, both MUSIC and RCM are almost incapable of detecting multiple targets, whereas ACGAN + SE + MD still achieves an F1-score of 0.28, significantly outperforming other deep learning models. Furthermore, its performance improves rapidly with increasing SNR, eventually reaching optimal levels. In summary, the proposed ACGAN + SE + MD model demonstrates outstanding comprehensive performance across all evaluation metrics, especially under low-SNR conditions, where it maintains strong robustness, high estimation precision, and reliable classification stability.

Experiment 4: Performance Comparison of Algorithms under Different Snapshot Numbers.

**Figure 8:**
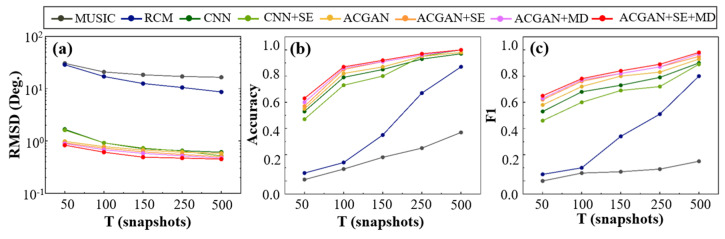
Evaluation of multiple algorithms under different snapshot settings. Subplots show (**a**) RMSE, (**b**) accuracy, and (**c**) F1-score across varying numbers of snapshots.

Figure 8 illustrates the changes in RMSE, Accuracy, and F1-score for various algorithms under different numbers of snapshots. Overall, with the increase in snapshots, the performance of all algorithms improves significantly. In Figure 8a, traditional algorithms show relatively high RMSE across all snapshot settings, indicating a strong dependency on data volume. In contrast, deep learning methods maintain relatively low RMSE even under small snapshot conditions. Among them, ACGAN + SE + MD achieves the best performance, reaching an RMSE of 0.83° at only 50 snapshots and gradually stabilizing as snapshots exceed 150. This indicates its robustness and insensitivity to snapshot variations. As shown in Figure 8b, traditional algorithms achieve accuracies below 0.1 when the number of snapshots is only 50, making them nearly ineffective. In contrast, CNN and ACGAN already exceed 0.5 accuracy at 50 snapshots, with ACGAN + SE + MD demonstrating a clear advantage. It consistently outperforms other methods across the entire range of snapshot numbers, particularly under low snapshot and low-SNR conditions. At 50 snapshots, ACGAN + SE + MD reaches an accuracy of 0.63, and quickly approaches optimal levels as the number of snapshots increases. This confirms that the model is capable of performing multi-target elevation classification even when information is limited, demonstrating strong generalizability and robustness to variations in snapshot count. In Figure 8c, the F1-scores of traditional methods remain low at small snapshot counts. Notably, MUSIC fails to recognize any targets effectively. In contrast, deep learning algorithms show significantly better identification capabilities across all snapshot ranges. ACGAN + SE + MD again outperforms other deep learning baselines, reaching an F1-score of 0.65 at 50 snapshots, and continues to improve as the number of snapshots increases. Compared with other models, the integration of the attention mechanism and multi-dilated convolution enhances the model’s perception of weak targets, significantly improving its sensitivity and discriminative power. In conclusion, the proposed ACGAN + SE + MD model demonstrates strong adaptability and application potential under low-snapshot conditions.

## 5. Discussion

The proposed model demonstrates stable elevation angle estimation performance under low-SNR and limited snapshot conditions. This robustness is attributed to the adaptive channel attention mechanism, which highlights key input channels, and the multi-scale dilated convolution module, which effectively captures complex features. Together, these components mitigate angular ambiguity commonly encountered in vertical array processing. Furthermore, adversarial training and the auxiliary classification branch enhance the model’s robustness and discriminative capability. However, it should be noted that the experiments were conducted on simulated data, and real-world validation in ocean environments has not yet been performed. Future work will involve applying the model to measured data to further assess its adaptability and generalization in complex acoustic conditions.

In terms of noise modeling, this study employed independent additive Gaussian noise on each channel to validate the robustness of the method under controllable conditions. However, real underwater acoustic noise and interference are more complex, including common types such as colored noise, spatially coherent noise, flow noise, and multipath effects. These factors may lead to non-white and non-independent noise scenarios, which can significantly affect direction estimation performance. In future work, we plan to incorporate more realistic noise models, such as shaping colored noise according to target power spectral density (PSD) curves, generating spatially correlated noise using covariance matrices, enhancing low-frequency flow noise, and modeling reverberation and multipath through delayed path superposition while preserving both phase and amplitude information, thereby improving model robustness in real scenarios.

In addition, this study simplified the modeling of source–array distance-dependent attenuation. The variation in source–receiver distance in the experiments (180–220 m) was small, leading to less than 2 dB of spreading loss, which is negligible compared to the analyzed SNR range. However, for larger-scale applications, the 6 dB per distance-doubling law and absorption loss should be explicitly considered to ensure consistency with free-field acoustics.

Finally, the large-scale synthetic dataset used in this study was generated through physics-based modeling of vector hydrophones. In real experimental conditions, obtaining such large-scale and accurately labeled data remains extremely challenging. Therefore, synthetic data should be regarded as a proxy for evaluating methodological performance rather than a replacement for real observations. In future work, we plan to integrate limited measured data with synthetic data through transfer learning or semi-unsupervised methods, in order to reduce the reliance on large-scale synthetic samples and better capture the variability of complex real acoustic environments.

## 6. Conclusions

This study proposes an ACGAN-based network architecture that integrates an attention mechanism and multi-scale features for the task of multi-target elevation angle estimation under low-SNR conditions. The model integrates an SE attention mechanism to emphasize the most informative input channels, and introduces an MDFA module to effectively extract salient features across a wide receptive field. This design addresses the challenge of angular ambiguity caused by target depth variation and interference in the vertical array response. In addition, the adversarial structure improves model robustness, while the auxiliary classification branch enables joint optimization, enhancing both decision-making capability and angular resolution. Simulation results demonstrate that the proposed model outperforms both traditional algorithms and existing deep learning approaches. Even under low-SNR and limited snapshot conditions, the model maintains strong elevation estimation accuracy and stability, effectively suppressing non-uniform noise and demonstrating excellent generalization.

## Figures and Tables

**Table 1:** RMSE of different algorithms under various SNR levels.

SNR (dB)	MUSIC	RCM	CNN	CNN + SE	ACGAN	ACGAN + SE	ACGAN + MD	ACGAN + SE + MD
−10	35.23	33.67	3.54	3.9	4.1	2.5	2.1	1.47
−7.5	31.02	28.47	1.88	1.52	1.71	1.14	1.05	0.86
−5	30.52	24.76	0.98	0.79	0.82	0.8	0.78	0.75
−2.5	28.59	17.68	0.74	0.61	0.63	0.65	0.61	0.52
0	16.5	8.66	0.61	0.52	0.58	0.5	0.48	0.45
2.5	8.34	5.76	0.52	0.45	0.45	0.43	0.42	0.4
5	5.37	3.68	0.47	0.42	0.39	0.39	0.39	0.36
7.5	3.25	2.98	0.41	0.4	0.38	0.32	0.3	0.28
10	2.57	1.96	0.38	0.36	0.35	0.28	0.26	0.25

## Data Availability

The data availability information has already been clearly stated in the manuscript, as this study is based on simulated data. No further changes are required for this section.
